# Editorial: Proceedings of the 3rd international conference on oral mucosal immunity and microbiome

**DOI:** 10.3389/froh.2024.1469116

**Published:** 2024-09-02

**Authors:** Georgios N. Belibasakis, George Hajishengallis

**Affiliations:** ^1^ Division of Oral Health and Periodontology, Department of Dental Medicine, Karolinska Institutet, Stockholm, Sweden; ^2^ Department of Basic and Translational Sciences, School of Dental Medicine, University of Pennsylvania, Philadelphia, PA, United States

**Keywords:** oral microbiome, oral mucosal immunity, oral disease, oral health, oral disease and systemic disease

**Editorial on the Research Topic**
Proceedings of the 3rd international conference on oral mucosal immunity and microbiome

**Figure 1:**
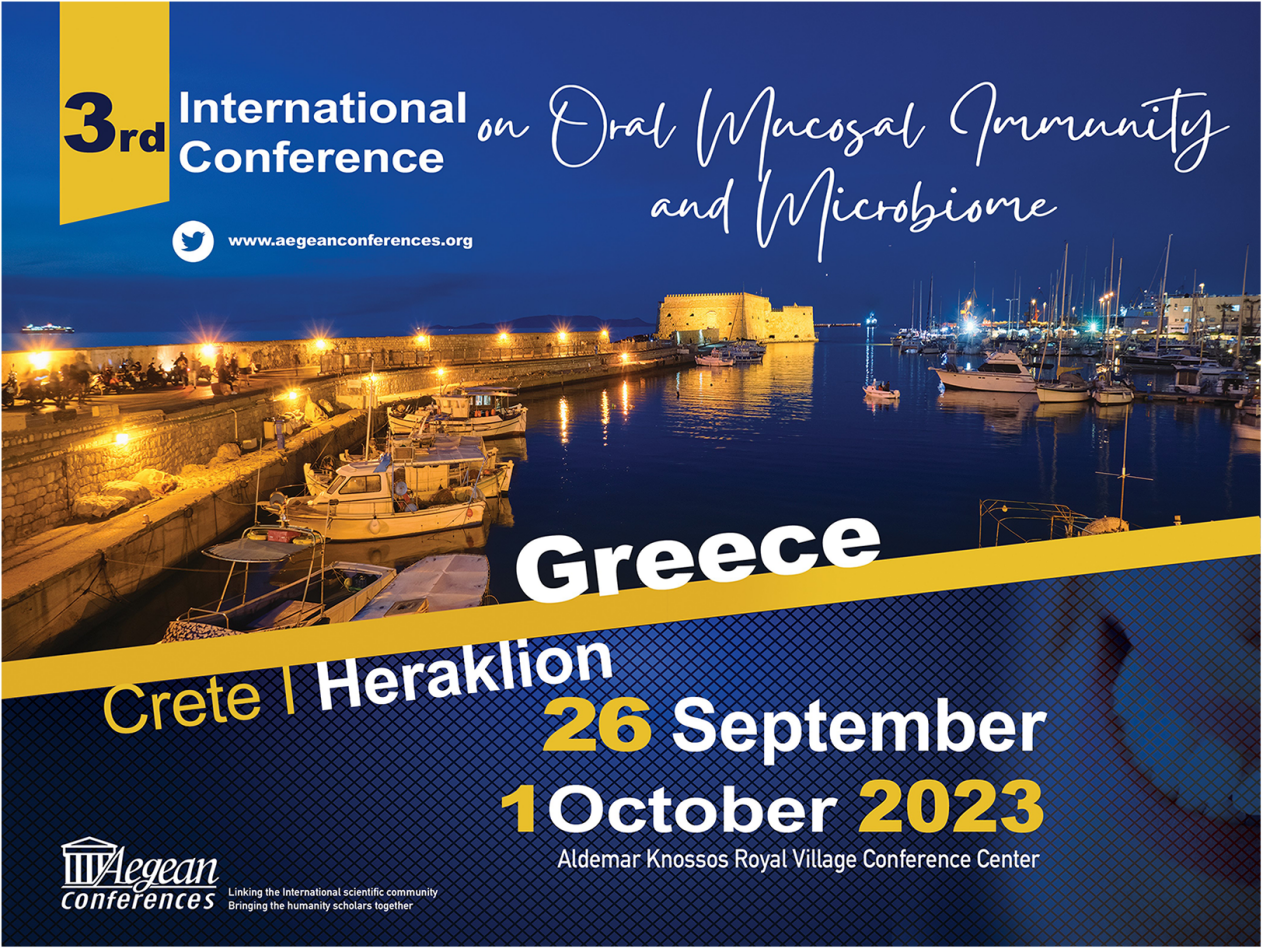
The poster announcement of the 3rd international conference on oral mucosal immunity and microbiome.

The International Conferences on Oral Mucosal Immunity and Microbiome held under the auspices of the Aegean Conferences, commonly designated as “OMIM,” have gained their place on the pedestal of top scientific events in dental and oral research. The present Research Topic in Frontiers in Oral Health reports the proceedings of the 3rd OMIM, which was held in Heraklion, Crete, Greece, from 26 September to 1 October 2023 (Figure 1). The conference addressed cutting-edge basic and translational research of oral immunological and microbiological scope, highlighting the importance of the composition and functions of the oral microbiome and the mechanisms underpinning its interactions with the host, particularly the oral mucosal immunity. Specific themes of the conference included microbial pathogenesis, inflammatory host response, polymicrobial communities, oral–systemic connection, and therapeutic approaches. In relation to previous OMIMs, we have achieved a greater integration of clinical translational research, with emphasis on the molecular determinants of the interconnection between oral and systemic diseases, as well as the molecular basis for novel therapeutic modalities.

**Figure 2:**
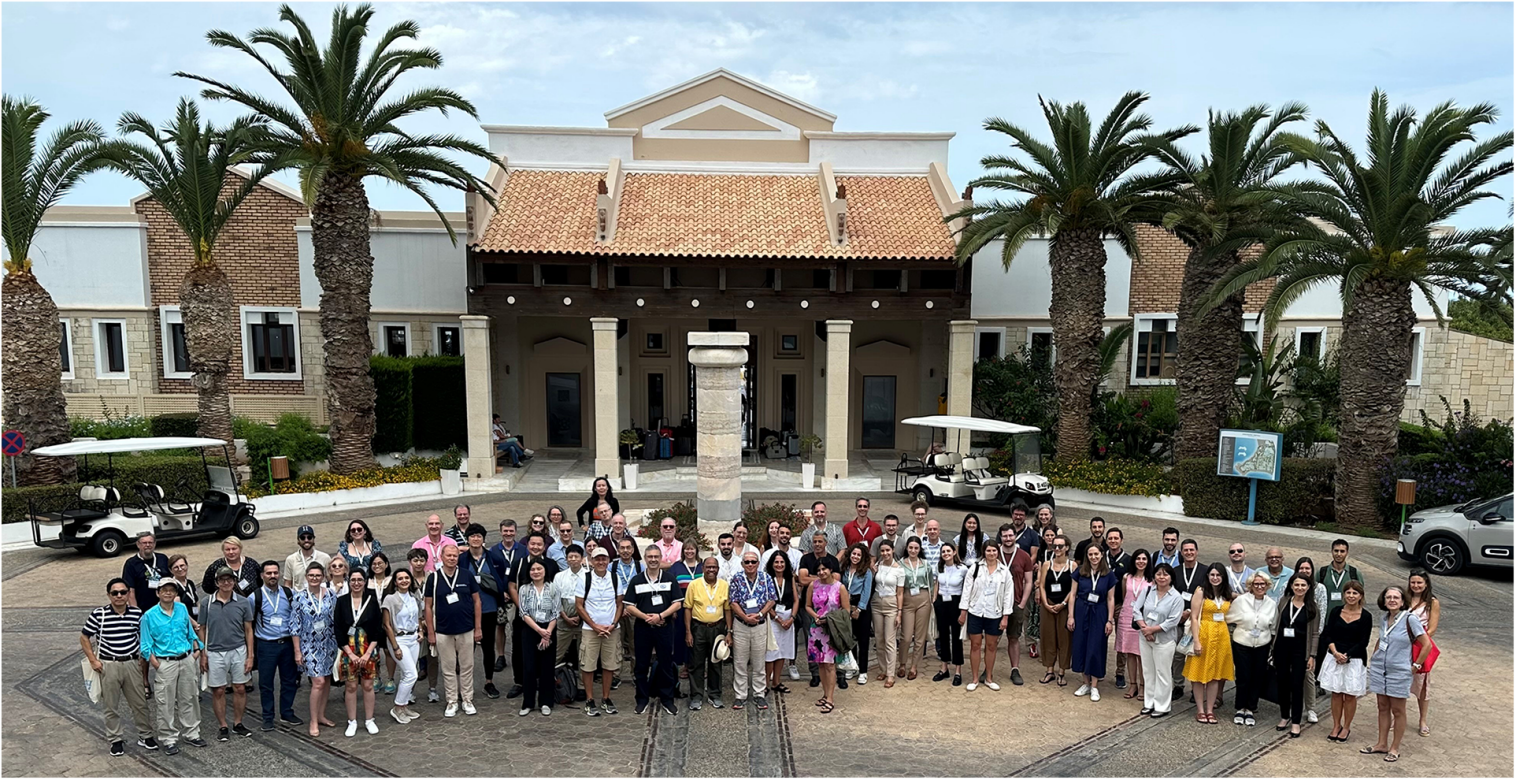
Group photograph of the 3rd international conference on oral mucosal immunity and microbiome participants.

The reports of the 1st and 2nd OMIM conferences have been published elsewhere (1, 2), whereas the detailed program of the 3rd OMIM conference can be found on the Aegean Conferences website, at the following link: https://www.aegeanconferences.org/src/App/sessions/165. A number of presentations at the 3rd OMIM conference (Figure 2) are featured in the form of short articles published in this designated Research Topic “*Proceedings of the 3rd international conference in oral mucosal immunity and microbiome*” and are highlighted in the present Editorial article.

An original report by Golda et al. addressed the antiviral activity of temporin-1CEb analogs against gingival infection with herpes simplex virus type 1 (HSV-1), a very widespread viral infection that poses increased risks in immunocompromised patients, especially due to the emergence of drug-resistant herpes strains. Novel temporin-1CEb analogs conjugated to peptides penetrating the host cell membrane were developed and demonstrated a significant reduction in viral replication in the oral mucosa. The peptide analogs target a heparan sulfate–mediated prohibition of HSV-1 cell adhesion and are effective in penetrating the gingival tissue to reach acyclovir-resistant strains.

In a review article, Malcolm and Culshaw discuss the well-established epidemiological links between rheumatoid arthritis and periodontitis and elaborate on recent data, shedding light on the mechanistic links that underlie these two diseases. The host–microbe interaction aspects and their subsequent adaptive immune response, such as antigen processing presentation and post-translational modification, are at the forefront of the plausible mechanistic links. Understanding these could help yield novel preventive and therapeutic strategies for both conditions.

In another review article, Choi discusses animal models for studying the pathogenesis and therapeutic interventions for oral lichen planus (OLP). This is particularly important since the treatment of recalcitrant erosive OLP is very challenging, raising an urgent need for specific pathway-targeted therapeutics. To date, only three animal models for OLP lesions have been reported and addressed in this review, along with a novel OLP mouse model first introduced at the 3rd OMIM conference. The validity of the available models is critically assessed and their potential future applications in OLP are discussed.

Kreth et al. addressed the concept of molecular commensalism, using the paradigm to examine how oral corynebacteria and their extracellular membrane vesicles shape microbiome interactions. Commensalism considers microbial diversity, rather than pathogen-centricity, in the conversion from health to disease and vice versa. Commensal microbes, such as certain streptococci and *Corynebacterium* spp. can counteract pathogenic species and modulate the host immune responses through hydrogen peroxide production and membrane vesicle secretion. Through such key functions, commensals can inherently suppress pathogens and bolster a symbiotic microbiome.

Higashi et al. addressed the paradoxical strategies of inflammophilic oral pathobionts to exploit innate immunity by manipulating neutrophil function. While an inflammatory response typically aims to inhibit or eradicate the ensuing infection, inflammophilic pathobionts not only survive this response but also gain a growth advantage within a rich inflammatory milieu. Some bacteria have indeed evolved inherent mechanisms that enable them to withstand or even utilize innate immune components to their existential advantage. In contrast, members of the commensal microbiome experience a competitive growth disadvantage under such inflammatory selective pressure. The review discusses examples of microbial-neutrophil interactions utilized by inflammophilic microbes to generate a positive feedback cycle that propagates dysbiotic chronic inflammation.

Lamont and Kuboniwa addressed the polymicrobial pathogenicity of *Porphyromonas gingivalis*. Polymicrobial communities are dependent on interspecies communication based on physical association, secreted small molecules, or nutritional exchange. While *P. gingivalis* is a periodontal pathobiont, the full expression of its virulence is effective when it is present in a polymicrobial community. Its multivalent fimbriae can mediate its attachment to other oral species, upon which they can initiate distinct transcriptional programs. *P. gingivalis* also responds to small molecules and nutritional cues produced by neighboring bacteria within a biofilm community. The established physiological interdependence between the cooperating microorganisms is the basis for the biofilm community to acquire an organismal entity with pathogenic potential.

Finally, Schäffer and Andrukhov addressed the interactive strategies of late colonizer *Tannerella forsythia* with the host. The type 9 protein secretion system and the O-glycosylation of its proteins are essential for the survival of *T. forsythia* in a biofilm and crucial for its immune evasion by the host. Its enzymatic virulence factors, including sialidase and proteases, potentiate its pathogenicity by degrading host glycoproteins and proteins, respectively. Its cell surface glycoproteins, such as S-layer and BspA, modulate the responses and adherence on the host cells, facilitating its colonization and tissue invasion capacities. The host cell target profile of *T. forsythia* is multifaceted and includes epithelial cells, neutrophils, macrophages, and mesenchymal stromal cells. In comparison to *P. gingivalis*, *T. forsythia* exhibits differential protease activity and host immune modulation patterns, suggesting distinctive roles in the pathogenesis of periodontitis.
